# Evaluation of Platelet Function Using Thromboelastography for a Patient With Pseudothrombocytopenia Requiring Cardiac Reoperation

**DOI:** 10.7759/cureus.28366

**Published:** 2022-08-24

**Authors:** Satoshi Kometani, Michihiko Kawai, Nami Teraoka, Reo Miyazaki, Hiroyuki Ikezaki

**Affiliations:** 1 Anesthesiology, Yamato Seiwa Hospital, Yamato, JPN; 2 Laboratory Medicine, Yamato Seiwa Hospital, Yamato, JPN; 3 Intensive Care Unit, Kawaguchi Cardiovascular and Respiratory Hospital, Kawaguchi, JPN

**Keywords:** low platelet count, transfusion practices, thromboelastography (teg), adult cardiac surgery, pseudothrombocytopenia

## Abstract

Pseudothrombocytopenia (PTCP) is a phenomenon in which platelet aggregation occurs in vitro when an anticoagulant such as ethylenediaminetetraacetic acid (EDTA) is used in a blood sample, causing automated cell counters (ACC) to calculate a lower platelet count than the actual count. While a peripheral blood smear is required to assess platelet count in PTCP accurately, such a time-consuming test is not accessible during the perioperative period. In this study, we evaluated platelet function using thromboelastography (TEG) for a patient with PTCP requiring cardiac reoperation. The preoperative TEG value of the patient was within the normal range, suggesting that TEG for PTCP reflects platelet function more accurately than ACC. Since there is an insufficient number of case reports on the use of TEG for PTCP, it is necessary to consider its usefulness not only during the perioperative period but also for other critical care.

## Introduction

Pseudothrombocytopenia (PTCP) is a phenomenon in which the structure of platelet glycoprotein (GP) IIb/IIIa changes when an anticoagulant such as ethylenediaminetetraacetic acid (EDTA) is used in a blood sample, leading to the development of blood immunoglobulins and antigenic antibody reactions that give rise to platelet aggregation in vitro and cause automated cell counters (ACC) to calculate lower platelet count than the actual value [1,2]. The frequency is estimated to be approximately 0.1% among blood cell count cases [3], so a peripheral blood smear is required for an accurate assessment. However, during the perioperative period of PTCP, evaluating platelet function with such a time-consuming test is not easy. Although thromboelastography (TEG), a point-of-care test, is considered useful, few reports have described the use of TEG for PTCP, and that too with limited findings [4,5]. In this study, we evaluated platelet function using TEG for a patient with PTCP requiring cardiac reoperation, along with a discussion on its usefulness thereof.

## Case presentation

A 73-year-old female (height 154 cm, weight 45 kg, body mass index 19 kg/m^2^) presented to our outpatient department with dyspnea on exertion. She had an American Society of Anesthesiologists (ASA) physical status of III. Past medical history was notable for post-double valve replacement (aortic valve/mitral valve) 10 years ago. Her current medications were warfarin, aspirin, and rosuvastatin.

Electrocardiogram findings and cardiac troponin values were normal. A transthoracic echocardiogram revealed severe mitral prosthetic valve stenosis with a mean gradient of 12 mmHg and severe aortic prosthetic valve stenosis with a peak gradient of 86 mmHg and a valve area of 0.48 cm^2^ with a left ventricle ejection fraction of 68%. Both valves had heavily thickened and calcified leaflets.

**Figure 1:**
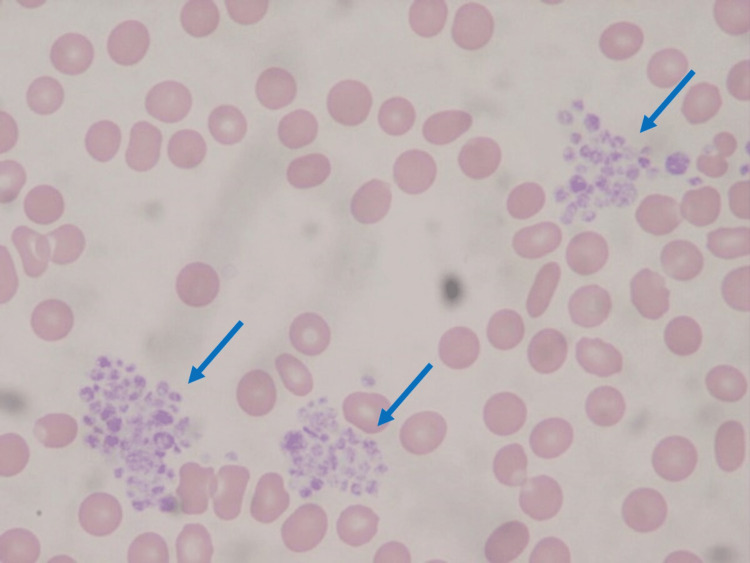
A peripheral smear in EDTA anticoagulated blood showing platelet aggregation (arrows) EDTA: ethylenediaminetetraacetic acid.

**Figure 2:**
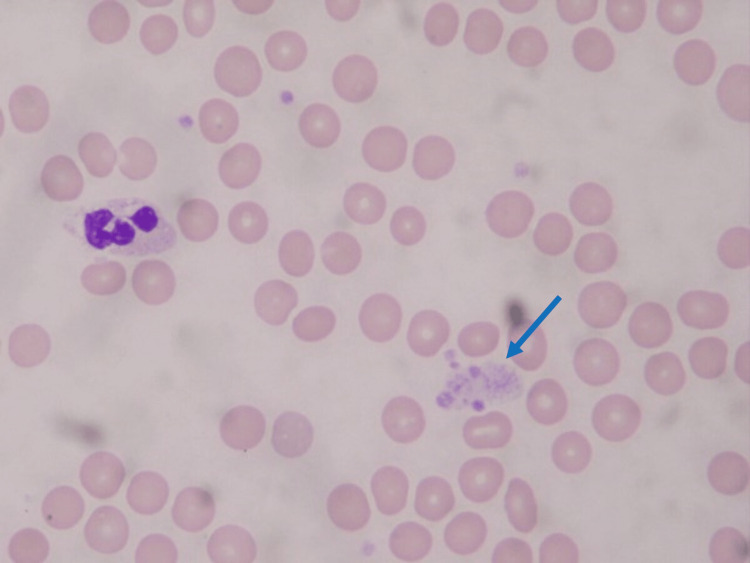
A peripheral smear in heparin anticoagulated blood showing platelet aggregation (arrow)

The patient was scheduled for an elective double valve reoperation. Her preoperative laboratory tests were normal, except for a decreased platelet count of 57×10^9^/L according to ACC using an EDTA-containing tube. Aspirin and rosuvastatin might have caused thrombocytopenia, but no punctate hemorrhage, mucosal bleeding, or splenomegaly was observed. When her blood was again collected and immediately counted, the platelet count increased to 110×10^9^/L. Upon a blood smear test, platelet aggregation was observed in both specimens containing EDTA and heparin (Figures 1, 2). Given the above findings, she was diagnosed with PTCP. Her IgG, IgM, and IgA levels were normal. A bone marrow examination was helpful for confirming whether impaired platelet production or accelerated platelet destruction had occurred, but it was not performed due to the procedure's invasiveness. Oral anticoagulation was discontinued before surgery.

As we expected massive bleeding during this surgery, we used TEG for a rapid and proper evaluation of platelet function. The blood specimen was collected in tubes containing citric acid and immediately examined by TEG®6s (Haemonetics Corporation, Boston, Massachusetts, United States). We referenced the maximum amplitude of citrated rapid TEG (CRT-MA) and the maximum amplitude of citrated functional fibrinogen (CFF-MA) to evaluate platelet function. In addition, estimated platelet counts from CRT-MA and CFF-MA values were calculated [6] and compared with the findings of the ACC using EDTA and heparin. The results are shown in Table 1.

**Table 1:** Perioperative laboratory findings and the TEG results EDTA: ethylenediaminetetraacetic acid; CRT-MA: maximum amplitude of citrated rapid TEG; CFF-MA: maximum amplitude of citrated functional fibrinogen; TEG: thromboelastography *Haemonetics Corporation, Boston, Massachusetts, United States

	Preoperative	After protamine administration	After platelet transfusion
Automated cell counter			
EDTA, platelet counts (×10^9^/L)	50	34	173
Heparin, platelet counts (×10^9^/L)	123	35	171
TEG6s*			
CRT-MA (mm, normal range 52-79)	62.6	32.2	58.9
CFF-MA (mm, normal range 15-32)	20.1	6.8	16.2
Estimated platelet counts (×10^9^/L)	158	27	160
Fibrinogen (mg/dL)	194	92	170
Hemoglobin (g/dL)	9.5	8.4	7.8
Activated clotting time (sec)	181	146	128

The preoperative TEG findings were within the normal range of CRT-MA 62.6 mm and CFF-MA 20.1 mm, with an estimated platelet count of 158×10^9^/L. In contrast, the platelet count with ACC was lower than the TEG estimated value of 50 × 10^9^/L in the EDTA tube and 123 × 10^9^/L in the heparin tube.

When 15,000 units (300 units/kg) of heparin were administered before the establishment of cardiopulmonary bypass (CPB), Activated clotting time (ACT) was prolonged from 181 seconds to 807 seconds. ACT was maintained above 400 seconds with additional heparin administration, as appropriate. The surgery was performed under normothermic conditions, and eight units of red blood cells and six units of fresh frozen plasma were transfused during CPB.

After completion of double valve replacement and cessation of CPB, 200mg of protamine was administrated, and ACT was shortened to 146 seconds. TEG at the exact moment was CRT-MA 32.2 mm and CFF-MA 6.8 mm, the estimated platelet count was 27 × 10^9^/L, and the platelet count of ACC was 34 × 10^9^/L in the EDTA tube and 35 × 10^9^/L in the heparin tube. We, therefore, deemed the patient to have true thrombocytopenia and transfused 20 units of platelets. After that, CRT-MA, CFF-MA, and estimated platelet count rose to 58.9 mm, 16.2 mm, and 160 × 10^9^/L, respectively. The platelet count of ACC was also confirmed to be 173 × 10^9^/L in the EDTA tube and 171 × 10^9^/L in the heparin tube, with the platelet function deemed adequate.

After transfusing an additional four units of red blood cells and six units of fresh frozen plasma, we confirmed surgical hemostasis and completed surgery. The anesthesia time was 525 minutes, the operation time was 460 minutes, and the CPB time was 307 minutes. She recovered well from surgery and was discharged on warfarin anticoagulation.

## Discussion

Thrombocytopenia is a hematologic pathology commonly found in ICU patients, with a prevalence of 8.3% to 67.6% [7]. It is also a frequent complication during the perioperative period, and bleeding is the most common cause. Understanding the dynamics of accurate platelet count and their temporal relevance is crucial, as platelet loss is informative about the magnitude of blood loss or trauma [8].

Other rare causes of thrombocytopenia are immune thrombocytopenic purpura (0.01%) and heparin-induced thrombocytopenia (0.3%), which are highly likely to be associated with the patient's mortality unless appropriately treated. Conversely, PCTP (0.1%) is asymptomatic and not associated with any bleeding or thrombosis abnormalities, making it unlikely that PTCP would be considered in the differential diagnosis of thrombocytopenia [8,9].

Although the autoantibodies of PTCP mainly consist of IgG, IgM and IgA have also been reported. It is presumed that platelet aggregation occurs when these autoantibodies react with glycoprotein (GP) IIb/IIIa, whose structure changes with the addition of anticoagulants [2]. However, there are many unclear points regarding the underlying mechanism, with approximately half of the antibodies unable to identify the trigger [10]. Since several reports on PTCP were discovered during the treatment of malignant neoplasms or cardiovascular diseases in ICUs, it has been pointed out that PTCP is likely to occur in critically ill patients [11]. The present patient also required a cardiac reoperation, so the previous cardiac surgery may have contributed to PTCP.

The CRT we referenced in the TEG procedure activates both the exogenous and endogenous coagulation system by adding kaolin and tissue factors to the citric acid-added blood sample and rapidly evaluates the maximum stiffness of the clot via the interaction between GP IIb/IIIa and fibrinogen in approximately 10 minutes at its maximum amplitude (MA). In addition, CFF evaluates the clot stiffness with fibrinogen alone by adding a GP IIb/IIIa antagonist to CRT and inhibiting platelet involvement. Estimating the platelet function and count is possible by subtracting this fibrinogen's contribution from the CRT [6]. However, to what extent blood samples from PTCP patients with citric acid added modify the TEG measurements is unclear. While PTCP is widely known to be EDTA-dependent, it also occurs with other anticoagulants such as citric acid and heparin in approximately 20% of cases, despite the weak aggregation degree [3,12]. We observed platelet aggregation in EDTA and heparin tubes in the smear test for this patient. Therefore, in the TEG measurement, there is a possibility that citric acid-dependent platelet aggregation occurred, so the results may have been modified by this factor.

The preoperative CRT-MA and CFF-MA in this patient were within the normal range, and the estimated platelet count of TEG did not decrease compared to that of ACC. The perioperative CFF-MA did not deviate from the value of serum fibrinogen, suggesting that TEG in PTCP more accurately reflects platelet function. Since platelet aggregation of PTCP progresses over time, it is recommended that collected blood specimens be analyzed promptly at 37°C [3]. In these regards, TEG, a point-of-care test, is considered suitable for PTCP. Since the number of reported cases concerning the use of TEG for PTCP is insufficient, we must further evaluate the usefulness not only during the perioperative period but also for other critical care.

## Conclusions

This case involved a patient with PTCP requiring cardiac reoperation using CPB, so it was assumed that the coagulation status during the perioperative period would dynamically fluctuate due to massive bleeding and blood dilution. TEG contributed properly by assessing platelet function rapidly and accurately to manage the patient's blood transfusion.

## References

[1] Shreiner DP, Bell WR (1973). Pseudothrombocytopenia: manifestation of a new type of platelet agglutinin. Blood.

[2] Casonato A, Bertomoro A, Pontara E, Dannhauser D, Lazzaro AR, Girolami A (1994). EDTA dependent pseudothrombocytopenia caused by antibodies against the cytoadhesive receptor of platelet gpIIB-IIIA. J Clin Pathol.

[3] Lin J, Luo Y, Yao S, Yan M, Li J, Ouyang W, Kuang M (2015). Discovery and correction of spurious low platelet counts due to EDTA-dependent pseudothrombocytopenia. J Clin Lab Anal.

[4] Chiba Y, Otsuka Y, Lefor AK, Sanui M (2022). Efficacy of point-of-care thromboelastography 6s to evaluate platelet function in a patient with pseudothrombocytopenia undergoing cardiopulmonary bypass: a case report. JA Clin Rep.

[5] Wilkes NJ, Smith NA, Mallett SV (2000). Anticoagulant-induced pseudothrombocytopenia in a patient presenting for coronary artery bypass grafting. Br J Anaesth.

[6] Tamura T (2019). Predicting results of fibrinogen and platelet levels by TEG6s during cardiopulmonary bypass: a pilot study. J Clin Anesth.

[7] Zarychanski R, Houston DS (2017). Assessing thrombocytopenia in the intensive care unit: the past, present, and future. Hematology Am Soc Hematol Educ Program.

[8] Santoshi RK, Patel R, Patel NS, Bansro V, Chhabra G (2022). A comprehensive review of thrombocytopenia with a spotlight on intensive care patients. Cureus.

[9] Williamson DR, Albert M, Heels-Ansdell D (2013). Thrombocytopenia in critically ill patients receiving thromboprophylaxis: frequency, risk factors, and outcomes. Chest.

[10] Sinha SK, Mandal PK, Mallick J (2011). Pseudothrombocytopenia -- a caveat. J Indian Med Assoc.

[11] Berkman N, Michaeli Y, Or R, Eldor A (1991). EDTA-dependent pseudothrombocytopenia: a clinical study of 18 patients and a review of the literature. Am J Hematol.

[12] Bizzaro N (1995). EDTA-dependent pseudothrombocytopenia: a clinical and epidemiological study of 112 cases, with 10-year follow-up. Am J Hematol.

